# Cancer associated macrophage-like cells and prognosis of esophageal cancer after chemoradiation therapy

**DOI:** 10.1186/s12967-020-02563-x

**Published:** 2020-11-04

**Authors:** Daniel J. Gironda, Daniel L. Adams, Jianzhong He, Ting Xu, Hui Gao, Yawei Qiao, Ritsuko Komaki, James M. Reuben, Zhongxing Liao, Mariela Blum-Murphy, Wayne L. Hofstetter, Cha-Mei Tang, Steven H. Lin

**Affiliations:** 1 Rutgers, The State University of New Jersey, 77 Hamilton Street, New Brunswick, NJ 08901 USA; 2 Creatv MicroTech Inc, Monmouth Junction, 9 Deer Park Dr, Potomac, NJ 08852 USA; 3 Department of Radiation Oncology, The University of Texas MD Anderson Cancer Center, 1515 Holcombe Blvd, Houston, TX 77030 USA; 4 Gastrointestinal Medical Oncology, The University of Texas MD Anderson Cancer Center, 1515 Holcombe Blvd, Houston, TX 77030 USA; 5 Thoracic and Cardiovascular Surgery, The University of Texas MD Anderson Cancer Center, 1515 Holcombe Blvd, Houston, TX 77030 USA; 6 Creatv MicroTech Inc, 9900 Belward Campus Dr, Rockville, MD 20850 USA

**Keywords:** Esophageal cancer, Cancer associated macrophage-like cell, Prognostic, Biomarker

## Abstract

**Background:**

Cancer Associated Macrophage-Like cells (CAMLs) are polynucleated circulating stromal cells found in the bloodstream of numerous solid-tumor malignancies. Variations within CAML size have been associated with poorer progression free survival (PFS) and overall survival (OS) in a variety of cancers; however, no study has evaluated their clinical significance in esophageal cancer (EC).

**Methods:**

To examine this significance, we ran a 2 year prospective pilot study consisting of newly diagnosed stage I-III EC patients (n = 32) receiving chemoradiotherapy (CRT). CAML sizes were sequentially monitored prior to CRT (BL), ~ 2 weeks into treatment (T1), and at the first available sample after the completion of CRT (T2).

**Results:**

We found CAMLs in 88% (n = 28/32) of all patient samples throughout the trial, with a sensitivity of 76% (n = 22/29) in pre-treatment screening samples. Improved 2 year PFS and OS was found in patients with CAMLs < 50 μm by the completion of CRT over patients with CAMLs ≥ 50 μm; PFS (HR = 12.0, 95% CI = 2.7–54.1, *p* = 0.004) and OS (HR = 9.0, 95%CI = 1.9–43.5, *p* = 0.019).

**Conclusions:**

Tracking CAML sizes throughout CRT as a minimally invasive biomarker may serve as a prognostic tool in mapping EC progression, and further studies are warranted to determine if presence of these cells prior to treatment suggest diagnostic value for at-risk populations.

## Background

Esophageal cancer (EC) is the eighth most common cancer histology in the world and sixth for highest mortality rate [1]. In the United States alone there were 17,300 cases of EC with 15,900 deaths in 2018 with an expected 17,700 new cases in 2019 and 18,440 cases in 2020 [2, 3]. Two major histology subtypes are associated with EC: (1) Adenocarcinoma (EAC), which comprises ~ 67% of the US patient population and (2) Squamous Cell Carcinoma (SCC), which comprises ~ 33% [2]. Over the past several decades, the EC 5 year survival rate has improved due to better staging and enhancements in cancer therapy; however, mortality rates remain high due to late diagnoses and delayed implementation of treatment [4, 5]. Previous studies have shown that ~ 30% of EC tumors are found after metastatic occurrence. Additionally, 5 year survival rates are 40% in patients with regional metastasis and 4% in patients with distant metastasis [6–9]. Delayed diagnoses are likely due to inaccuracies in standard endoscopic techniques used for finding early stage tumors [10]. Furthermore, it is common for early malignancies to display themselves as macroscopically healthy under endoscopy, and there is a lack of expert pathologists that can properly identify these abnormalities shown in endoscopic images [11]. Early detection of EC is crucial for determining patient treatment plans and improving patient progression free survival (PFS) and overall survival (OS), yet due to the delayed presence of patient symptomology (i.e. Dysphagia, rapid weight loss) physicians cannot start standard of care accordingly [12]. In order to enhance patient prognosis in early stage EC, new diagnostic methodologies are needed to ensure early intervention.

Currently, the standard of care for EC is determined based on the location of the tumor, the patients’ medical fitness, and the stage in which it is diagnosed. Patients with resectable localized tumors are typically treated with preoperative neoadjuvant chemoradiotherapy (CRT) or chemotherapy alone, followed by surgery [13]. In contrast, patients with unresectable tumors are treated with definitive CRT alone [6, 14, 15]. Despite clinical improvements in the treatment for EC, both SCC and EAC 5 year disease free survival (DFS) remains limited to only 39.8% in the United States [16]. The ability to monitor a patient’s tumor response throughout treatment may allow for more precise adjustments to therapeutic regimes to optimize the management of EC disease.

Using liquid biopsies for screening and monitoring cancer has the advantage of being non-invasive and having the ability to sequentially test at multiple time points for determining response to therapy. Circulating Tumor Cell (CTC) analysis is an FDA approved method for blood-based monitoring for the prognosis of cancer patients; however, its use is limited by CTC rarity in non-metastatic disease and CTC scarcity in EC (18–27%) [17, 18]. Circulating tumor DNA (ctDNA) is a newer biomarker that identifies mutated tumor DNA in whole blood and can possibly screen for early stage EC, however, ctDNA is found in only 20% of Stage I EC and ctDNA is not prognostic for determining PFS or OS during treatment [19]. Recently ctDNA was shown to have a sensitivity of 60% in newly diagnosed EC pretreatment samples, with 71% sensitivity over 3 months after the completion of standard CRT; more sensitive assays need to be developed for better quantification and analysis of ctDNA [20]. Autoantibodies and other cancer-related protein biomarkers (i.e. Fas ligand, NYO-ESO-1, etc.) derived from patient’s blood serum have also shown promise for the early detection and monitoring of EAC, yet no definitive biomarkers have been standardized [21]. Epigenetic protein biomarkers (i.e. p21, p53, CRP, and Hb) are all potentially prognostic in EC, however validation studies with consistent methodologies (i.e. dose administration, timeline of use) are still needed [22]. Predicting patients’ prognoses via blood based biopsies, epigenetics, and autoantibodies still lack validity in locally advanced EC, requiring further investigation into potential progression biomarkers to improve patients’ treatment plans and outcomes [23].

CAMLs are a recently identified cancer specific circulating stromal cell common in a variety of solid cancers regardless of disease stage [24, 25]. A range of CAML sizes have been identified as 21–300 μm in length, with median sizes for CAMLs, CTCs, and normal WBCs being 43.5, 18.8, and 12.4 μm, respectively [25, 26]. Initial studies on CAMLs as a prognostic tool for cancer progression in localized lung cancer has been described [27]. This initial study of EC suggests that monitoring CAML changes throughout therapy might predict treatment response [26, 27]. To date, no study has evaluated the presence of CAMLs or their clinical utility in EC. To better understand the clinical utility of CAMLs in EC, we initiated a prospective pilot study in patients (n = 32) with locally advanced EC to evaluate CAML sizes throughout CRT treatment to compare overall patient prognosis and possible clinical utility. We sought to determine if sequential monitoring of CAMLs could act as a blood-based biomarker to screen for EC, and further, if CAMLs provide predictions in the progression of disease.

## Materials and methods

### Patient recruitment

Thirty-two stage I–III esophageal cancer patients were recruited in this 2 year prospective pilot study (Table 1). Anonymized peripheral blood samples were collected in accordance with MD Anderson Cancer Center’s local Institutional Review Board (IRB) approval and with patients’ informed consent. Patients were recruited from July 2013 until June of 2014 with baseline BL sample taken ~ 1–4 weeks after pathological confirmation of EC, but before starting standard CRT treatment. Time points T1 and T2 were collected halfway through treatment, ~ 2 weeks, and at the first available sample after the completion of CRT (approximately 6 weeks after the start of treatment), respectively. Patients’ randomized and anonymized blood samples (7.5 mL) were collected into CellSave preservative vacutainer tubes (Menarini Silicon Biosystems) and prepared according to standard operating procedures at MD Anderson (see details below). Purified slide specimens were then shipped to Creatv MicroTech Inc. clinical laboratory for cell enumeration and analysis. Results between institutions were blinded and not shared nor communicated until the study was completed.

**Table 1:** Patient population and known clinical parameters

Patient demographics	n=32
Age (years)	Median = 64. 5 (44–76)
Sex	
Male	29 (91%)
Female	3 (9%)
Race	
White	30 (94%)
Black	1 (3%)
Hispanic	1 (3%)
Tumor histology	
Adenocarcinoma	25 (78%)
Squamous cell	7 (22%)
Tumor grade	
G1	1 (3%)
G2	14 (44%)
G3	17 (53%)
cT category	
cT1	1 (3%)
cT2	3 (9%)
cT3	27 (85%)
Unknown	1 (3%)
eN category	
cNO	11 (34%)
cN1	13 (41%)
cN2	6 (19%)
cN3	1 (3%)
Unknown	1 (3%)
eM category	
cMO	32 (100%)
cM1	0 (0%)
cTNM stage	
lb	1 (3%)
lc	1 (3%)
IIa	4 (12%)
lib	6 (19%)
lila	14 (44%)
lllb	5 (16%)
Unknown	1 (3%)
RT modality	
Proton	9 (28%)
IMRT	18 (57%)
30	1 (3%)
VMAT	4 (12%)
Surgery	17 (53%)
Induction chemo	10 (31%)

### Isolation of CAMLs

Peripheral whole blood samples (7.5 mL) were collected and filtered using a CellSieve™ Microfiltration assay on a low-pressure vacuum system. CellSieve™ Microfiltration assays isolate CAMLs and other cancer-associated circulating cells ≥ 7 μm by size exclusion [25, 27, 28]. Specifically, a CellSieve™ microfilter is washed with PBS and centered onto a filter holder. 7.5 mL of whole blood is prefixed with an equal amount of Prefixation Buffer for 20 min and then is filtered by a CellSieve™ microfilter to collect large cells in ~ 3 min. Filters are washed. Then cells are post-fixated for 15 min and then permeabilized for 15 min. The cells are stained with an antibody mixture of Cytokeratins 8, 18 & 19 tagged with FITC, EpCAM tagged with AF555, and CD45 tagged with Cy5. After staining, filters are washed and then mounted with Fluoromount-G with DAPI (Southern Biotech). CAMLs, CTCs, and Epithelial to Mesenchymal Transition cells (EMTs) are determined by cell morphology and phenotypic expression of CD45, EpCAM, Cytokeratins 8, 18, 19, and DAPI; as previously described [25, 28]. CAMLs were identified by their large size of 21–300 μm, DAPI positive polyploid nuclei, and often express CD45. White blood cells were identified based on their relatively small size, mononucleated appearance, and high expression of CD45 and DAPI. CTCs were identified based on their size, mononucleated appearance, and high expression of cytokeratin in filamentous pattern and no expression of CD45. Enumeration of cancer-associated circulating cells was performed with an Olympus BX54WI Fluorescent microscope with Carl Zeiss AxioCam and Zen 2011 Blue (Carl Zeiss) by a trained cytologist. Denucleated CAMLs, apoptotic CTCs, and EMTs were not included in our enumeration analysis.

### Statistical analysis

Unblinding and initial data analyses were done independently at both MD Anderson and Creatv MicroTech. Final analyses were done using MATLAB R2013A using counts and CAML sizes from each respective cancer associated cell subtype taken from the known patient population of 32 patients. CAML counts of “0” were included into our statistical analyses for determining mean max CAML sizes, stratifying patient groupings based on cell size, and evaluating survival outcomes by including CAML sizes of 0 μm into the < 50 μm group. One patient (3%) dropped off study and was censored before the end of the 24 month trial. Baseline (BL) blood samples were collected prior to induction of any therapy. Follow up samples (T1) were defined as ~ 18 days (range = 9–28 days) after the start of treatment, or (T2) immediately after the completion of CRT (ranging 22–100 days). Samples were available for 91% of patients at BL (n = 29/32), 75% of patients at T1 (n = 24/32), and 59% of patients at T2 (n = 19/32). Kaplan–Meier plots were determined by log-rank analysis with significance being defined as *p* value < 0.050 and trending < 0.150. Two-sided univariate analyses were run using all known clinical variables (Table 1, Additional file 1: Tables S1 and S2). Multivariate Cox proportional-hazards analysis was used to evaluate 1) time to progression and 2) time to death while accounting for potentially significant risk factors (Additional file 1: Table S3).

## Results

### Patient population and circulating cell presence

In total, 32 stage I-III newly diagnosed untreated locally advanced EC patients were recruited prior to receiving standard of care CRT, with CRT regimes averaging 37 days of radiation. A minimum of one CAML was found in 88% (n = 28/32) of patient samples, including all available time points throughout treatment.This study’s cohort consisted of 91% (n = 29/32) male and 9% (n = 3/32) female patients (Table 1). A patient cohort containing 9% females is slightly lower than the expected EC incidence rate for women (~ 22%), and is a potential limitation for the results of this pilot study. EAC accounted for 78% of patients (n = 25/32) and SCC 22% (n = 7/32). Clinical stage distribution was found as such, 3% (n = 1/32) in stage Ib, 3% (n = 1/32) in stage Ic, 12% (4/32) in stage IIa, 19% (n = 6/32) in stage IIb, 44% in stage IIIa, 16% (n = 5/32) in stage IIIb, and 3% (n = 1/32) of unknown stage with no evidence of metastasis.

**Fig. 1:**
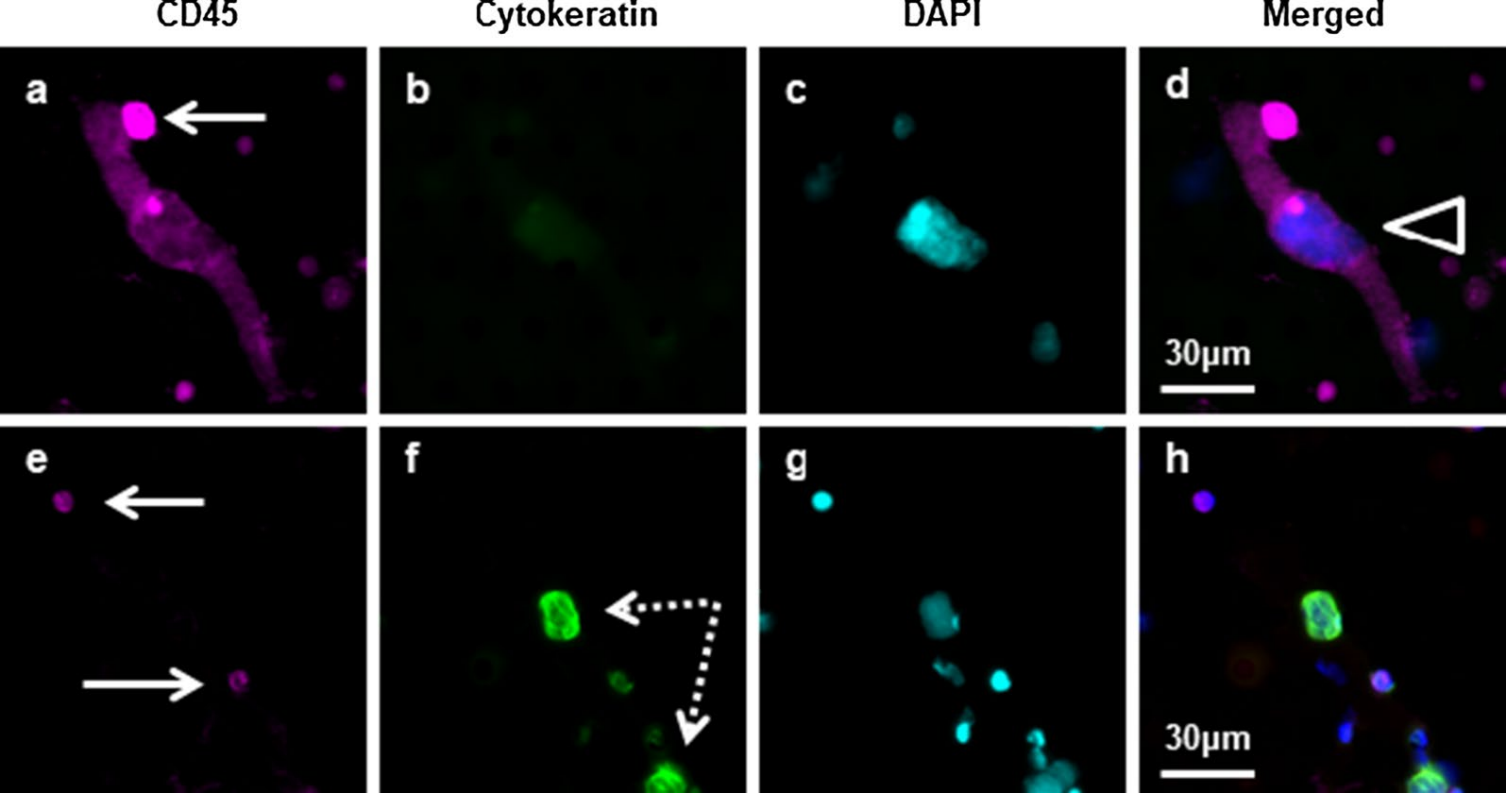
Example of a CAML, normal white blood cells (WBCs) and circulating tumor cells (CTCs) with size comparisons. Cells were stained with an antibody mixture of CD45 (purple), cytokeratin (green), and DAPI (light blue). **a**–**d** A CAML(white open triangle) which was -65 m in length and CD45 + & DAPI + . CAML was attached to a normal WBC − 10 m in length (white arrows) which appears CD45 + and DAPI + . **e**–**h** CTCs (white dashed arrows) are Cytokeratin + & CD45−.CTCs shown near to normal WBCs (white arrows) that are CD45 + and DAPI + .Normal WBCs are typically − 8–10 m cells highly expressive of CD45.CAMLs are large, typically CD45 + ,low Cytokeratin + , with polynucleated DAPI nucleus. CTCs are CD45-with high filamented Cytokeratin signal

In order to evaluate the relationship between CAML size and patient outcomes, PFS and OS based on CAML sizes ≥ 50 or < 50 μm were compared at BL, T1 and T2. The enumeration of CAML cells found an average of 6.5 CAMLs/7.5 mL of blood throughout all known time points, with an average CAML size of 47 μm. During treatment it was found that the average CAML size in patients initially increased after induction of radiation from 34 to 49 µm, and then decreased to 45 µm after completion of radiation; Fig. 3 and Additional file 1: Table S1. This would indicate an inflammatory immune response to treatment that eventually dissipates with tumor shrinkage. Though it was determined that overall CAML average was not predictive for survival outcomes, the presence of any large CAML was a significant prognosticator for worse outcome. Including all 77 available time points used in this study, CAML monitoring via microfiltration had a fail rate of 3% (n = 2/77) due to clotting of the blood. CAML identification was based on a large cell diameter, polyploid nuclei, and high expression of CD45. In addition to CAMLs, CTC presence was found in only 12.5% of the samples (n = 4/32) throughout all available time points, with two patients presenting stage IIa, one patient stage IIb, and the fourth patient of unknown stage. CTCs were identified by their mononucleated appearance, high expression of cytokeratin, and lack of CD45 expression. To compare the differences between circulating cancer-associated cells**, **Fig. 1 portrays a size and stain comparison between a CAML, CTC, and a regular white blood cell.

### Sequential CAML monitoring

**Fig. 2:**
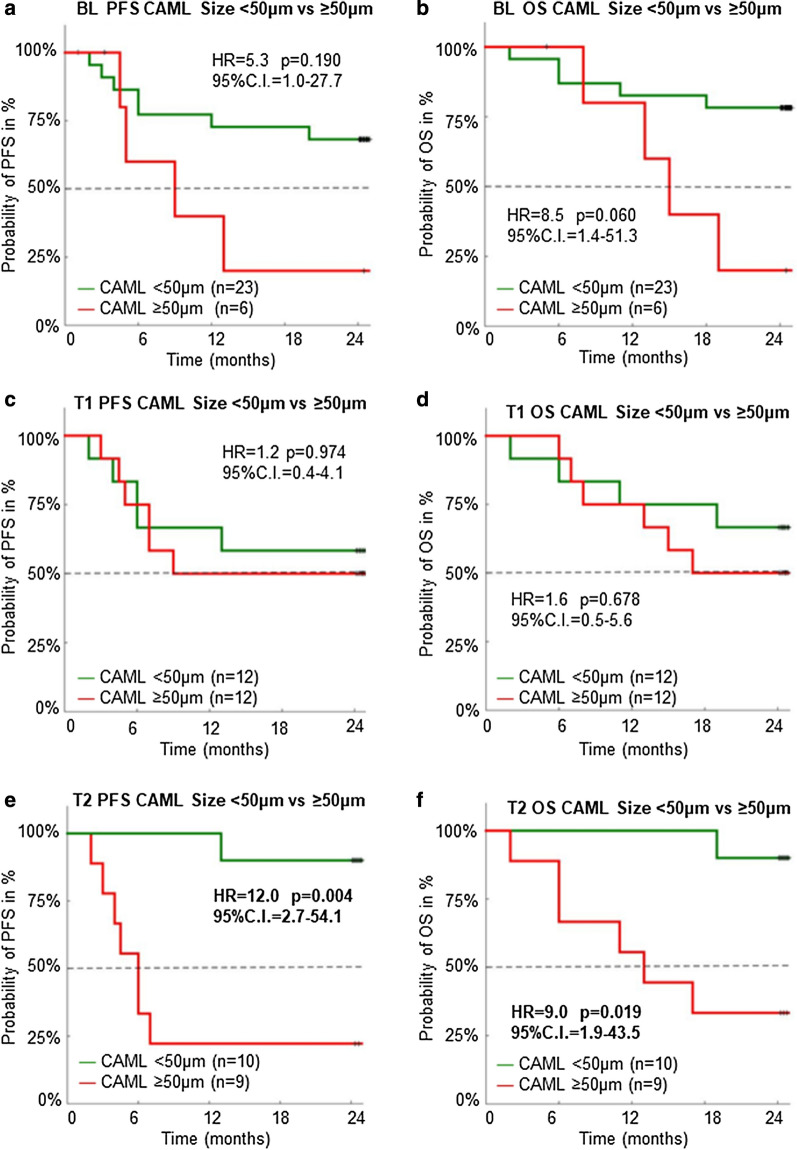
Kaplan–Meier Survival Estimates of CAML Size at BL, T1 and T2. **a**, **c**, and **e** Demonstrate patient PFS outcomes throughout treatment. **b**, **d**, and **f** Show patient OS outcomes throughout treatment

At BL, prior to the start of CRT (n = 29), we found an average of ~ 5 CAMLs/7.5 mL of blood, with a minimum of one CAML seen in 76% (n = 22/29) of available baseline samples. The average max CAML size seen among patients at BL was found to be 38 μm. When comparing survival outcomes based on CAML size, patients with CAML sizes < 50 μm (n = 23) had non-significant trends toward improved PFS compared to patients with CAML sizes ≥ 50 μm (n = 6) (HR = 5.3, 95% CI = 1.0–27.7, *p* = 0.190). (Fig. 2a) Similarly, OS at BL showed that patients with CAML sizes < 50 μm trended toward improved survival over patients with CAMLs ≥ 50 μm (HR = 8.5, 95% CI = 1.4–51.3, *p* = 0.060) (Fig. 2b**)**.

At T1, the midpoint of radiation therapy, we found an average of ~ 10 CAMLs/7.5 mL of blood, with an average max CAML size of 58 μm, and CAMLs were found in 96% (n = 23/24) of samples. Despite most samples having CAMLs at this time point, CAML size was not a significant prognostic indicator when comparing the < 50 μm group (n = 12) versus the ≥ 50 μm group (n = 12); PFS (HR = 1.2, 95% CI = 0.371–4.068, *p* = 0.974) and OS (HR = 1.6, 95%CI = 0.5–5.6, p = 0.678). (Fig. 2c,d) At this time, these initial findings indicate that there appears to be no statistical clinical significance of CAML size and presence at T1_._

At T2, the first sample taken after the completion of radiation therapy, we found an average of ~ 5 CAMLs/7.5 mL of blood and an average max diameter of 49 μm was identified. In all available samples at this time point, CAMLs were found in 89% (n = 17/19) of patients. We found that patients with < 50 μm CAMLs had significantly improved PFS and OS when compared patients with ≥ 50 μm CAMLs, PFS (HR = 12.0, 95% CI = 2.7–54.1,* p* = 0.004) and OS (HR = 9.0, 95% CI = 1.9–43.5, *p* = 0.019). (Fig. 2e, f, Additional file 1: Table S2). Interestingly, analysis of average CAML presence, average CAML size, and average max CAML size found a linear correlation between the CAML averages after completion of CRT (i.e. T2) and the pathological stage of the patients (Additional file 1: Table S1). Multivariate Cox proportional-hazards analysis determined that engorged CAMLs at the T2 time point 1) trended toward being a significant independent predictor for worsened PFS (*p* = 0.0505) and 2) were a significant independent predictor for shortened OS (p = 0.0407); Additional file 1: Table S3. However, given the small patient population in this analysis (n = 19) expanded patient population is necessary.

### Patterns found in locally advanced EC

**Fig. 3:**
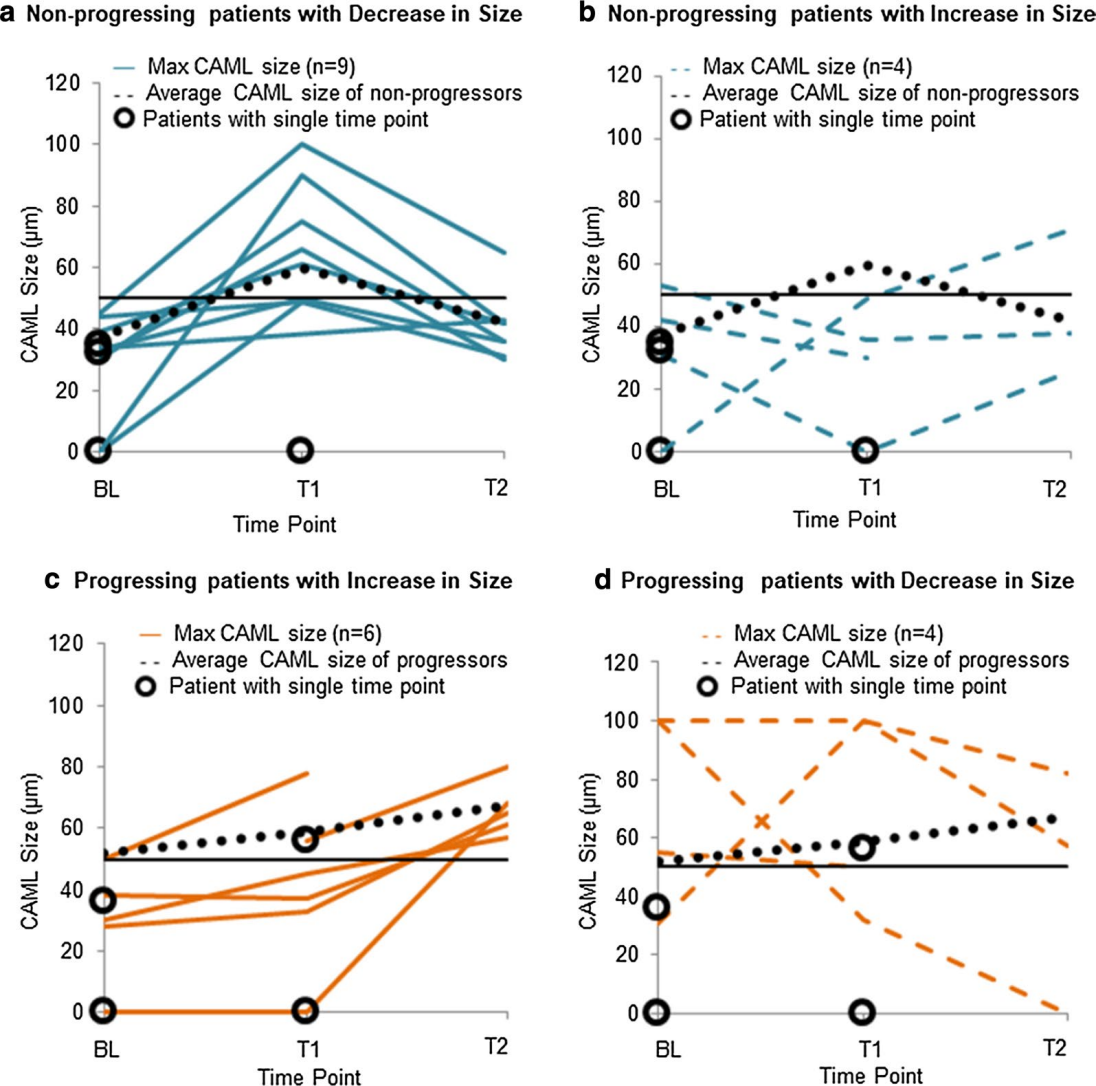
Changes of CAML sizes in non-progressing or progressing patients before, during and after induction of CRT. Average trends in the largest CAML size were visualized by averaging all patients that did not progress within 24 months (**a**, **b**) or all patients that progressed within 24 months (**c**, **d**) black dotted lines. **a**, **b** 70% of patients that did not progress within 2 years had an increase in macrophage engorgement during CRT, with a decrease in engorgement after treatment completion. **c**, **d** 60% of patients that progressed within 2 years had a gradual increase in macrophage engorgement at every time point during CRT with an overall increase in after treatment completion. Patients with a single time, i.e.no sequential data available, are indicated by open circles

To better understand the stratification of survival based on CAML size, we evaluated the two primary patient populations- patients that eventually progressed within 24 months and patients that did not progress within 24 months (Fig. 3). After completion of radiation (T2), 85% (n = 11/13) of non-progressing patients had smaller CAMLs (< 50 μm). In contrast, only 10% (n = 1/10) of patients that progressed had smaller CAMLs (< 50 μm). Using 24 months as an endpoint, this would equate to a prognostic accuracy of 87% in predicting disease recurrence based on the presence of large CAMLs after completion of radiation.

Further evaluation of CAML trends during treatment seemed to identify two general patterns in CAMLs engorgement. In patients that eventually progressed, average CAML sizes increased from 51.7 μm (BL), to 58.7 μm (T1), and 67.1 μm (T2). This demonstrated a linear growth in CAML engorgement from baseline to the completion of CRT. In patients that did not progress average CAML sizes increased from 37.4 μm (BL) to 58.5 μm (T1). However, by T2 average CAMLs decreased to 41.8 μm. In total while most patients (71%) had an increase from BL to T1, patients, however in the patients that progressed there was an additional 14% increase in CAML size at T2. In contrast, in patients that did not progress there was a 29% decrease at the T2 blood sampling. While further analyses in a larger patient population will be needed to confirm these trends in CAML growth in relation to PFS and OS, this pattern does suggest a biological and clinical difference in a patient’s immunological response to radiation treatment. 

In addition to CAML analysis, patients’ OS and PFS were analyzed based on EC histology and whether or not they had received surgical resection (Table 1). When comparing the two histological types, we found that patients with EAC (n = 25) trended for improved PFS over SCC (n = 7) (HR = 3.9, 95% CI = 0.91–14.8, *p* = 0.149). Interestingly, OS was significantly in favor for EAC (HR = 14.8, 95% CI = 2.63–83.33, *p* = 0.009). In line with previous studies, patients that had received surgery post-completion of standard CRT trended toward improved PFS compared to patients that did not undergo surgery, PFS (HR = 2.8, 95% CI = 0.12–1.04, *p* = 0.104) but OS did not appear different (HR = 2.5, 95% CI = 0.12–1.32, *p* = 0.228).

## Discussion

EC is widely known for its increased rates of post-treatment progression and high mortality [14]. Early detection of EC and prompt treatment is crucial for extending patient PFS and OS, however, delayed diagnoses due to inaccurate endoscopic tools and image misreads are still common. At this time, there are no reliable biomarkers available for the rapid detection and prediction of patient progression in locally advanced EC. In this prospective pilot study, we examined patients with locally advanced EC to determine the clinical significance of CAMLs for treatment response before, during and after therapy induction.

We monitored patients’ CAML sizes prior to treatment (BL), ~ 2 weeks into treatment (T1), and ~ 4–8 weeks after the completion of radiation (T2), comparing CAML size and number for patterns of clinical significance. CAMLs were present in 76% of local/locally advanced EC patients prior to treatments indicating their possible use as an EC biomarker, and engorged CAML sizes found at the BL time point may be indicative of more aggressive disease subtypes. At mid-treatment with radiation (T1), CAMLs did not provide insight on efficacy of treatment, though it appeared that a biological response was identified as most patients had an increase in CAML number and size. After completion of definitive CRT (T2), these data suggest that patients with large CAML sizes (≥ 50 μm) are indicative of disease recurrence within 2 years of initial treatment. While these results must now be expanded upon and validated, the sequential monitoring of disease using simple blood draws may identify more aggressive EC disease subtypes and used for monitoring patient treatment in locally advanced EC.

While not significant, but in line with previous works, this data suggests that patients with larger CAMLs in their blood prior to the start of treatment non-significantly trends towards worse outcomes. With a larger cohort of patients, it seems possible that these findings may reach clinical significance. This implies that CAML sizes taken from baseline draws may indicate patients with more aggressive disease, and that these patients may not respond to standard of care. Our non-progressing patients show a common pattern of low CAML sizes at baseline, an immunological flare spike in CAML size at T1, and a decrease in CAML size by T2. Progressing patients typically showed a gradual increase in CAML size along each time point during treatment. By examining these common progression patterns, it may be possible to determine how patients are responding to treatment, which can lead to the modification of treatment regimens to maximize patient outcomes.

Prior studies on macrophage involvement in the EC microenvironment have shown increased pro-inflammatory response and improved tumor invasion after initiation of radiation treatment [29, 30]. These studies would suggest that the fluctuations of CAML engorgement may be indicative of a positive response in non-progressing EC patients and may signify beneficial patient response to treatment. To date, the exact mechanisms of CAML engorgement and intravasation into circulation is still unknown. It may be possible that the patterns found here are caused by the constant flux of phagocytic macrophages into the tumor microenvironment during treatment. Activation of the immune system via recognition of the tumor may be indicated by large CAML size increases, as macrophages could be phagocytosing newly recognized tumor neoantigens caused by tumor death. By actively monitoring CAML size in response to treatment, clinicians may be able to identify positive changes in a patients’ immune response and recognize effective treatment. In contrast, the lack of CAML size increases may signify a lack of patient immunological response, and indicate a need for a different therapy. The active monitoring of CAML sizes throughout CRT may help determine patient immunocompetency, and the lack of an immunological response halfway through CRT could signify the need for second line immunotherapies in order to activate the immune system.

## Conclusion

Overall, we found that patients with CAML sizes ≥ 50 μm at the completion of standard CRT are at a higher risk of disease progression compared to patients with CAMLs < 50 μm. As a minimally invasive procedure, CAML diagnostics obtained through sequentially taken liquid biopsies may open the doorway to finding a consistent biomarker that aids in the detection of early stage EC and helps predict metastatic progression. Prior studies on liquid biopsies testing for epigenetic biomarkers and serum based protein assays have shown promise in the detection of EC, yet no consistent methodologies and accurate results have been found. Moving forward, CAML size analysis taken from peripheral whole blood can be run in conjunction with plasma ctDNA, or protein testing which may increase the sensitivity and accuracy in predicting prognoses in EC. In combination, the use of CAMLs as a biomarker to actively monitor and adjust treatment plans based on sequential monitoring may provide insight throughout standard of care and suggest second line therapy intervention when a lack of immunological response is identified.

## Supplementary information

**Additional file 1.** Supplementary tables.

## Data Availability

All datasets used and/or analyzed throughout this study are available from the corresponding author based on sensible request.

## Abbreviations

CAML: Cancer Associated Macrophage-Like cell
PFS: Progression Free Survival
OS: Overall Survival
EC: Esophageal Cancer
CRT: Chemoradiotherapy
BL: Baseline
HR: Hazard Ratio
CI: Confidence Interval
EAC: Esophageal Adenocarcinoma
SCC: Squamous Cell Carcinoma
DFS: Disease Free Survival
CTC: Circulating Tumor Cell
ctDNA: Circulating Tumor DNA
WBC: White Blood Cell
IRB: Institutional Review Board
EMT: Epithelial to Mesenchymal Transition cells
