# Supplementary figures and images for: Cancer associated macrophage-like cells and prognosis of esophageal cancer after chemoradiation therapy
Source: J Transl Med. 2020 Nov 4;18:413. doi: 10.1186/s12967-020-02563-x (PMC7640696; doi:10.1186/s12967-020-02563-x)

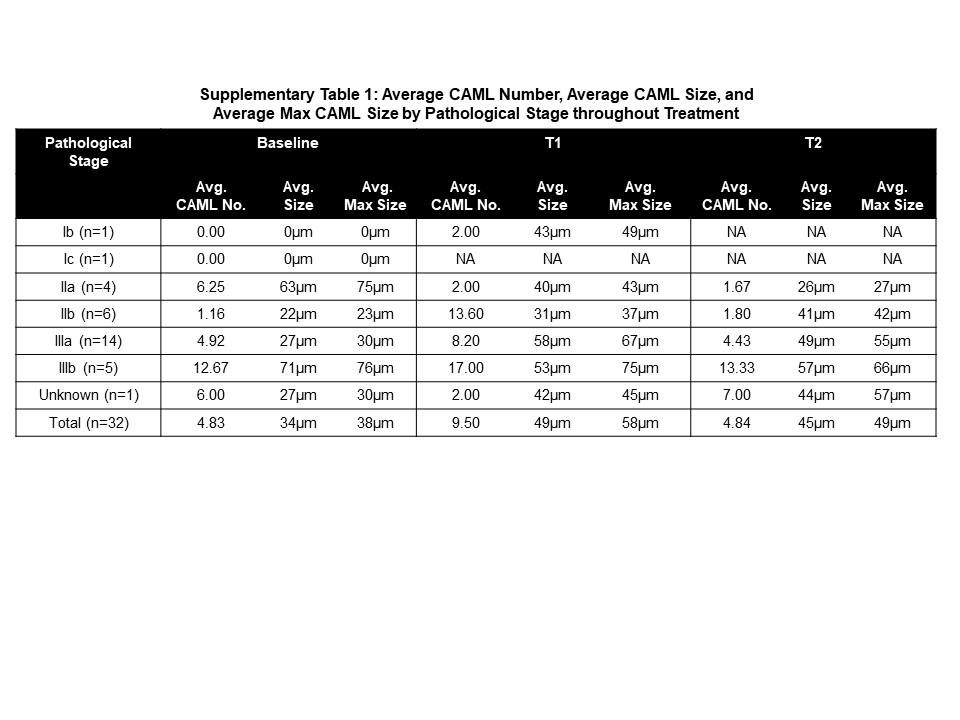


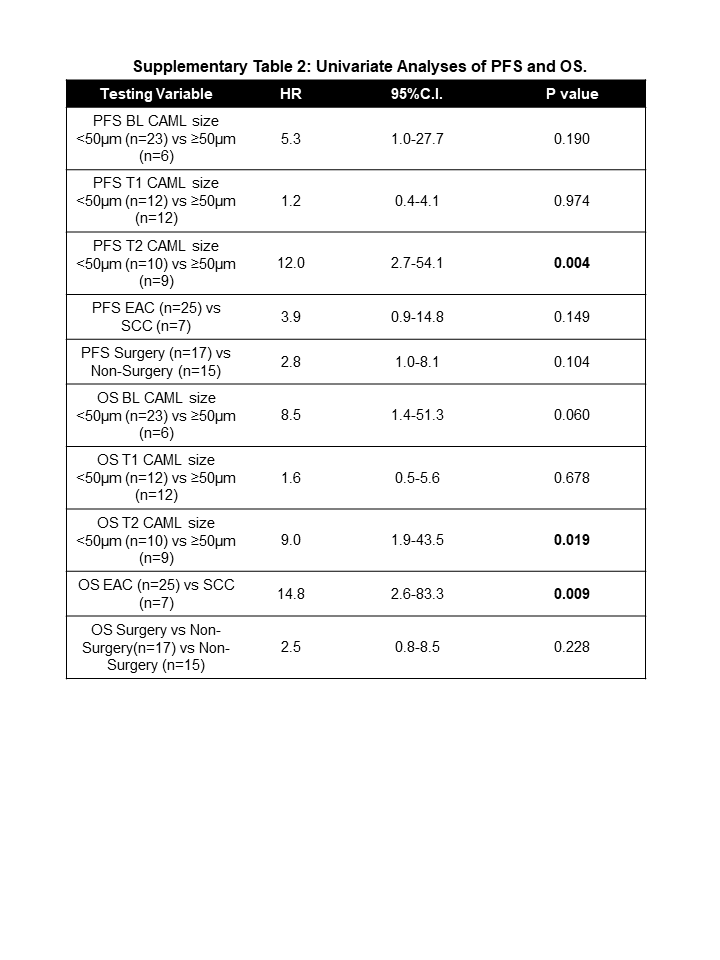


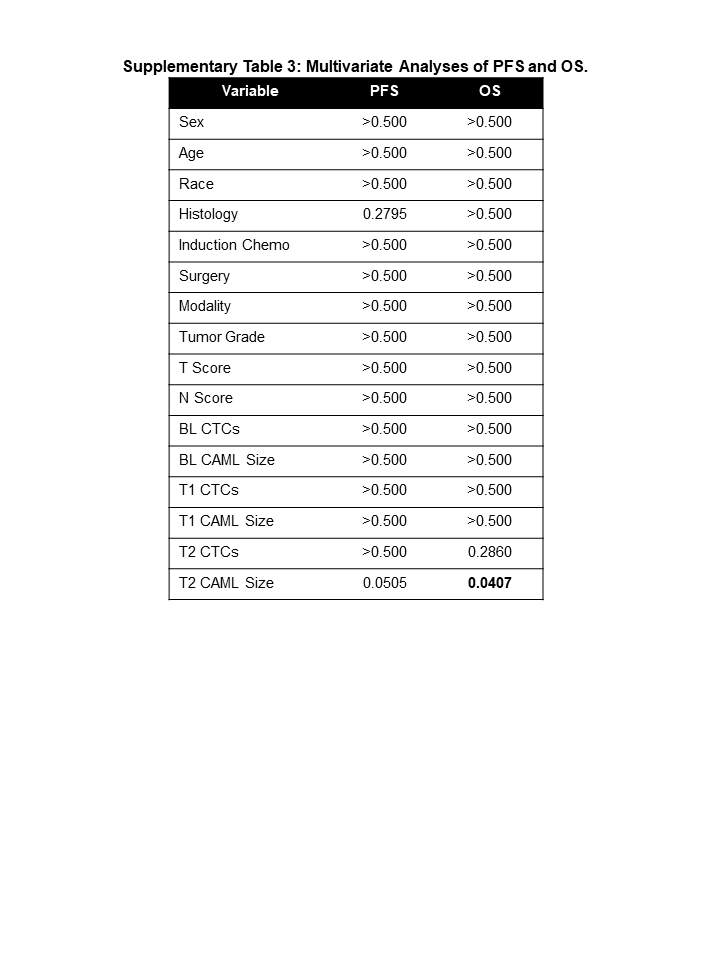

Supplement: Supplementary file 1 — Additional file 1. Supplementary tables. [file 12967_2020_2563_MOESM1_ESM.docx]
